# The physical attributes of sub-elite rugby union referees of inland provinces in South Africa

**DOI:** 10.17159/2078-516X/2021/v33i1a8835

**Published:** 2021-02-10

**Authors:** C Le Roux, A Lombard, A Green

**Affiliations:** Department of Sport and Movement Studies, Faculty of Health Sciences, University of Johannesburg, South Africa

**Keywords:** field testing, match officials, performance, morphology

## Abstract

**Background:**

The purpose of the on-field referee is to implement the laws of the game. For the referee to do this successfully, he is required to keep up with the pace of play.

**Objectives:**

The aim of this study was to determine the physical attributes of sub-elite rugby union referees of inland provinces in South Africa.

**Methods:**

A total of 82 referees (age: 26.5 ± 6.4 years; stature: 177.3 ± 6.8 cm; body mass: 79.1 ± 14.7 kg) were assessed with a reliable testing battery.

**Results:**

The participants showed a refined aerobic capacity (VO_2max_: 61.8 ± 11.0 mL·min^−1^·kg^−1^) and good agility (Illinois Agility Test: 17.2 ± 3.8 s). A two-way unbalanced ANOVA was performed for all referees’ attributes between qualification levels (levels 1–4) and union affiliations (three unions) at a significance level of p<0.05. The results yielded significant differences across the three unions in age (p=0.002), Yo-Yo distance (p=0.0001), aerobic capacity (p=0.0001), plank time (p=0.0001) and agility (p=0.027). Similarly, differences were reported across the four qualifications in aerobic capacity (p=0.0001) and agility (p=0.037).

**Conclusion:**

These differences may be due to the diverse training programmes offered by the various unions. Additionally, an increased level of physical fitness may occur when progressing up the qualification levels.

Rugby union is contested by two teams, each consisting of 15 players. The objective of the game is to outscore the opponent by means of tries, penalties and conversions. The laws of the game are interpreted and applied by an on-field referee and two assistant referees.^[1]^ The on-field referee is primarily responsible for officiating the entirety of match play, including player behaviour, imposing penalties and adjudicating the competition; whilst the touch judges and assistant referees assist with matters relating to the side-lines, in-goal play, including attempted goal kicks and reporting foul play.^[2]^ The panel of referees can be expanded for higher competitive level matches to include fourth and fifth assistant referees who regulate match substitutions and other duties.^[3]^

The on-field referees are required to keep up with play and to position themselves in favourable positions to make appropriate decisions. As such, they must attain similar movements and velocities to the rugby players. Rugby players cover on average 5 800 m during a game, interspersed between bouts of various velocities and intensities. This indicates that for 80 minutes the players require a variety of aerobic and anaerobic capacities.^[4–8]^ Moreover, player demands include physical collisions and largely static efforts, such as rucking and scrummaging.^[9]^ However, unlike the players, referees are not required to participate in physical contests, but are required to maintain similar physical efforts to the players.

Referees play a pivotal role in match progression and outcome. They need to interpret a massive amount of information to make fast, clear and correct decisions about the game.^[5]^ A referee needs to manage a game in a unique environment, influenced by the quality of the game, the attitude of the players and spectators, while ensuring peak physical performance. The referee undertakes high-intensity running in open play and must be visible, without interfering with a team’s progress.^[7]^ Once open play has stopped (tackle, ruck, maul), the referee will need to slow down and be in a position to make an appropriate call. It is therefore essential that they are able to slow down and change direction to where they can optimally locate the ball. The total average distance covered by referees during a rugby match is usually 6 400 m.^[3]^ Of the total distance, approximately 881 s is spent standing still, 736 s walking, 346 s jogging, 139 s sprinting and lastly, 372 s spent moving laterally.^[6]^ Referees covered the majority of the distance at velocities of between 0.51–2.00 m.s^−1^ and 2.01–4.00 m.s^−1 [3]^, interspersed with efforts at higher velocities, thus demonstrating the intermittent nature of the required movement patterns during the game.^[6]^ Cunningham et al. reported that there are no significant differences in the running performances for referees from the first half to the second half, although the first 10 minutes consisted of the most intense work of all the 10 minute periods in the game.^[11]^ Bester and colleagues^[8]^ reported that referees had an average heart rate of 186.5 beats per min with an eight percent difference in heart rate between the two halves. These findings indicate that rugby refereeing is highly intense, placing high demands on a referee’s aerobic and anaerobic energy systems.^[12]^ Thus for 80 minutes a referee will need good aerobic capacity and the ability to stay mentally focused for that duration of time to make accurate decisions.

However, the physical demands of players and referees during a game may not be equal, and different physical and physiological parameters are required. Furthermore, these differences between players and referees may require different training requirements. This study aims to establish the physical attributes of sub-elite rugby referees, highlighting the physical preparation that the referee goes through to meet the demands of the game and their anthropometric values.

## Methods

This was a descriptive cross-sectional study determining the physical attributes of sub-elite referees. Sub-elite referees were defined as referees who adjudicate amateur level rugby and do not serve on the South African Referee Union Panel. Three unions were selected for this study based on their proximity to the research institution. Two major rugby unions from Gauteng and one from the North West Province were selected. A sample of 82 referees from three unions (age: 26.5 ± 6.4 years; stature: 177.3 ± 6.8 cm; body mass: 79.1 ± 14.7 kg and body fat percentage: 10.9 ± 3.2%) with an average of 2.8 ± 1.7 years’ experience volunteered and signed the informed consent form to participate in this study. All ethical procedures were reviewed and approved by the Faculty of Health Sciences Ethics Committee (REC -01-153-2017), University of Johannesburg.

### Procedure

Body mass and stature were assessed with an electronic scale (813 Seca, Hamburg Germany) and stadiometer (213 Seca, Hamburg Germany). Body fat percentage (Harpenden Sussex, England) was calculated using the sum of six skinfolds method as proposed by Davies et al.^[9]^ Flexibility was assessed by means of a standard sit-and-reach box^[10]^ to the nearest millimetre. Aerobic capacity was estimated using the Yo-Yo Intermittent Recovery Test^[11]^ and agility by means of the Illinois Agility Test.^[12]^ Upper body muscle endurance was assessed using the one minute push-up test^[10]^ and the plank hold for the same time.^[13]^ The standing long jump test^[14]^ (or broad jump test) was used to estimate lower limb muscular power. All procedures were conducted in a single training session at the rugby union’s training facilities. This testing protocol has many similarities to the BokSmart study ensuring that it relates to the demands of South African rugby.^[14]^

### Data analysis

The sample was divided into the three unions and the respective qualification levels. Qualification levels are ranked from one to four, with one being the highest amateur referee ranking level. The higher qualified referees have completed more World Rugby officiating courses. Due to upper body injuries on the day of testing, the sample sizes for the various tests had some discrepancies in the number of referees participating

### Statistical analysis

Data are presented as mean ± standard deviation. The Shapiro-Wilk test was performed on each variable to determine their distributions. An unbalanced two-way ANOVA (qualification level x union affiliation) was conducted for each variable across the affiliated unions and qualification levels, followed by Bonferroni post hoc tests. All statistical analyses were performed using SPSS 24 (IBM), and significance was defined at a level of p < 0.05.

## Results

Anthropometric and physical performance tests for the 82 rugby referees are reported in Table 1. For age, a large significant interaction (p=0.0001) was identified between the referees’ qualification level and union affiliation (Table 2). In addition, significant differences were noted between referee affiliations (unions) for age (p=0.002), Yo-Yo distance (p=0.0001), estimated VO_2max_ (p=0.0001), plank time (p=0.0001), but not in their qualification level (age: p=0.131; Yo-Yo distance: p=0.087; estimated VO_2max_: p=0.106; plank time: p=0.389).

Referees affiliated with Union 1 were older than those affiliated with Union 2 (p=0.01) but not Union 3 (p=1.00). Of the three unions, referees affiliated with Union 2 covered the lowest distance in the Yo-Yo Intermittent Recovery Test (p=0.0001) and subsequently had the lowest estimated VO_2max_ (p=0.0001). Regarding plank performance, referees associated with Union 2 recorded the lowest times compared to those related to Union 1 (p=0.0001) and Union 3 (p=0.0001).

The results from the two-way ANOVA (Table 2) indicated a significant interaction for the time taken to complete the Illinois Agility Test by referees between their qualification and union (p=0.029). Additionally, there were significant differences in the Illinois Agility Test’s completion times between unions (p=0.027) and qualification (p=0.037). Referees affiliated with Union 1 outperformed referees affiliated with Union 2 (p=0.006). Furthermore, referees with qualification Level 1 outperformed those with qualification Level 3 in the Illinois Agility Test (p=0.035). No further differences were noted between the affiliated unions or qualification levels in the Illinois Agility Test (Table 2). Lastly, there were no significant differences for height (p=0.778), weight (p=0.150), body fat percentage (p=0.164), sit-and-reach (p= 0.828), push-up (p= 0.045) and the broad jump (p=0.425) (Table 2).

## Discussion

The study aimed to establish the physical attributes and performance parameters of sub-elite rugby referees of inland provinces in South Africa. A sample group of 82 referees were tested and subsequently divided into registered unions and qualification levels to investigate any potential differences. With regard to the unions and qualifications, there were differences in age, aerobic capacity, agility, and muscle endurance.

Referees are required to keep up with play to determine the outcome of the game and make important decisions about what they see in front of them during the game. Therefore, one would expect referees to have similar in-game demands as players. In comparison to rugby union referees, rugby players run 500 m less per game than rugby union referees.^[4]^ Rugby players averaged 1 506 m in the Yo-Yo Intermittent Recovery Test compared to the rugby referees who averaged 2 910 m.^[15]^ However, one component exclusive to players is that of frequent collisions^[16]^ Rugby players are about 18 kg heavier and 19 cm taller than most referees.^[9]^ Over recent years, rugby players have become heavier and taller, possibly indicating the importance of collisions and physical contact in modern rugby.^[11,17]^ However, the requirements of refereeing versus playing are vastly different. As such, comparisons will be conducted with non-contact football codes.

Interestingly, when comparing rugby union referees with football referees, football referees run 4 000 m more per game.^[18]^ This could be due to the time differences between the football codes, field dimensions and different running patterns required. It is noted that football referees are about seven kg lighter and have about two percent less body fat^[19]^ than the rugby union referees presented in the current study. Lastly, elite rugby union referees covered on average 8 030 m^[7]^ per game which is significantly more than the sub-elite referees.^[3]^ This could be attributed to the playing levels and physical condition differences between the referees. A reduced mass and body fat percentage may be advantageous, allowing referees to effectively cover greater distances. Indeed, elite referees have been reported to have similar body masses (83.4 kg) and body fat percentages (11.6%), compared to those reported in the current study.^[8]^ Additionally, the difference in anthropometrics between referees in both codes may be due to many of the referees being ex-players. Irrespective of the in-game demands, referees require refined physical and physiological attributes to perform.

Rugby referees will have repeated efforts of running bursts with times to rest during the stoppages in games.^[4]^ Therefore, the Yo-Yo Intermittent Recovery Test is an appropriate test for rugby referees as it closely matches the running demands of the game.^[15]^ Rugby referees in this study averaged 2 910 m in the Yo-Yo Intermittent Recovery Test, which is the same distance as that for football referees.^[20]^ The Yo-Yo Intermittent Recovery Test can be used to estimate the participant’s VO_2max_. The total sample estimated VO_2max_ was 61.8 mL·min^−1^·kg^−1^ compared to the soccer referees’ 50.2 mL·min^−1^·kg^−1^ on the treadmill and 48.6 mL·min^−1^·kg^−1^ during the field tests.^[21]^ This substantial difference could be due to the two different test protocols used to determine the VO_2max_. The two protocols used were the Yo-Yo Intermittent Recovery Test estimation versus direct lab measurements on the treadmill.^[21]^

The ability to rapidly accelerate, decelerate and change direction is essential for the referees to keep up with the demands of the game. If referees are competent with these skills they will be in a better position to adjudicate the game optimally. When comparing rugby and football referees, they performed the same, namely, 17.2 seconds to complete the Illinois Agility Test.^[22]^ A standing broad jump can be used to estimate lower limb power. Professional football referees outperformed rugby referees in the current study by 10% in the standing broad jump. Those football referees were professional referees^[19]^ which could be the main reason for the difference in performance, as the rugby sample group are amateurs.

Differences across teams, leagues and playing levels in players and referees is well documented.^[23]^ The current study investigated these differences by dividing the whole sample into three groups, based on their affiliated unions. There are marked differences in how the game is played at international level between Northern Hemisphere and Southern Hemisphere countries.^[24]^ Likewise, differences can be seen in the same sporting code but playing in different leagues.^[25]^ A similar difference can be expected inter-provincially and between different unions in the same country. Several physical performances were reported to be different across the three unions namely, the Yo-Yo Intermittent Recovery Test, estimated VO_2max_, the Illinois Agility Test, and the muscle endurance tests. These differences between unions could be due to union training preferences, education, and the overall level of the game being played in that union. Specifically, referees from Union 2 were the youngest, which could be due to the fact that they come from a university town and had just started their refereeing career. Union 2 also covered the least distance in the Yo-Yo Intermittent Recovery Test thus resulting in the worst score on the estimated VO_2max._ Lastly, Union 2 also performed worst in the plank hold test compared to the other two unions. As previously mentioned, Union 2 was the youngest of the unions resulting in less experience, a younger training age, and the lowest understanding of what is expected from them physically.

Referees should be assessed numerous times during a year to give them an appropriate level of competency and to monitor their physical fitness levels. Level 1 referees are more highly qualified compared to those in Level 4. The more highly qualified referees completed more courses such as the World Rugby Level 2 officiating course and performed better on the yearly written and fitness exams. It would seem as though unions have a large influence over the physical abilities of referees. That is, the results from the current study indicated many differences in the physical capabilities with the Yo-Yo Intermittent Recovery Test, the Illinois Agility Test and plank hold tests. These differences could indicate the different unions’ implementation of training protocols, testing and physical preparedness for referees. Additionally, the intensity of matches at the various union levels may be different, depending on the leagues in which they compete. A similar trend is seen with athletes across various codes.^[26]^ It is widely reported that physical performances increase with an athlete’s playing level.^[27]^ Thus, the data reported in the current study show that qualification levels in rugby referees are analogous with an athlete’s playing level. The more experienced referees all had the highest qualification levels. These referees performed the best in the Illinois Agility Test, indicating that the ability to change direction is critically important. This could mean that the referees’ qualification levels and experience may provide them with greater physical preparation for games, as they are more aware of the matches’ demands. However, further detailed investigations are required.

## Conclusion

For referees to keep up with the demands of the game, they require high aerobic capacity, muscular endurance and strength. If the referee can refine these physical attributes, they can position themselves better leading to more accurate decisions in the game. This will ensure that it is played fairly, and make it enjoyable for all. There are many differences between the unions related to the parameters tested in this study. Thus the different standards between the unions, may be due to the incompatible education or training programmes offered by the unions. An improved level of fitness should occur when progressing up the qualification levels similar to the changes seen in the players, but more research is required on this.

## Figures and Tables

**Table 1:** Anthropometric and physical performance characteristics of 82 rugby union referees

Characteristic	Mean ± SD
Height (cm)	177.3 ± 6.8
Body mass (kg)	79.1 ± 14.7
Body fat (%)	10.9 ± 3.2
Sit and Reach (cm)	30.8 ± 9.5
Yo-Yo Intermittent Recovery Test (m)	2910 ± 750
Estimated VO_2max_ (ml/kg/min)	61.8 ± 11.0
Illinois Agility Test (s)	17.2 ± 3.8
Push-ups (Repetitions)	39 ± 6
Plank (s)	145 ± 40
Standing broad Jump (m)	2.1 ± 0.3

**Table 2:** Anthropometric and physical performance characteristics of 82 rugby union referees compared across union affiliation and qualification level

Characteristic	Qualification level	Union 1	Union 2	Union 3	Total for qualification levels
**Age (years)** †*#	1	27.2 ± 6.3	26.6 ± 5.3	26.5 ± 2.8	26.6 ± 5.3
	2	28.7 ± 4.8	28.3 ± 7.5		28.6 ± 5.4
	3	24.2 ± 9.4	22.4 ± 5.5		23.3 ± 7.4
	4	40.0 ± 0.0	19.7 ± 3.0		24.2 ± 9.3

	**Total for unions**	27.7 ± 7.0	23.7 ± 5.4	26.5 ± 2.8	26.1 ± 6.2

**Height (cm)**	1	178.6 ± 5.7	177.7 ± 8.4	175.2 ± 5.3	177.7 ± 6.4
	2	175.4 ± 7.1	180.2 ± 6.7		177.0 ± 7.0
	3	176.4 ± 8.8	174.5 ± 6.6		175.5 ± 7.4
	4	173.5 ± 6.0	178.8 ± 7.3		177.6 ± 7.0

	**Total for unions**	177.5 ± 6.3	177.7 ± 7.4	175.2 ± 5.3	177.3 ± 6.6

**Weight (kg)**	1	80.1 ± 9.9	81.5 ± 10.0	78.2 ± 11.0	80.1 ± 10.0
	2	79.3 ± 13.8	82.1 ± 3.9		80.2 ± 11.2
	3	72.8 ± 12.8	79.9 ± 15.9		76.4 ± 14.1
	4	67.1 ± 7.7	83.4 ± 22.9		79.7 ± 21.3

	**Total for unions**	78.3 ± 11.1	81.8 ± 14.3	78.2 ± 11.0	79.6 ± 12.3

**Body fat (%)**	1	10.6 ± 3.6	10.9 ± 1.9	10.6 ± 2.8	10.7 ± 3.0
	2	14.0 ± 5.3	10.3 ± 1.3		12.7 ± 4.6
	3	9.0 ± 2.3	9.7 ± 2.8		9.3 ± 2.4
	4	12.3 ± 3.4	9.6 ± 1.7		10.2 ± 2.2

	**Total for unions**	11.0 ± 3.9	10.2 ± 1.9	10.6 ± 2.8	10.7 ± 3.2

**Sit and Reach (cm)**	1	31.6 ± 10.6	29.0 ± 11.7	32.4 ± 8.2	31.1 ± 10.3
	2	29.9 ± 7.8	22.7 ± 3.2		27.5 ± 7.3
	3	30.1 ± 7.8	34.4 ± 9.0		32.3 ± 8.2
	4	27.0 ± 4.2	30.9 ± 6.5		30.0 ± 6.1

	**Total for unions**	30.9 ± 9.5	29.8 ± 9.6	32.4 ± 8.2	30.7 ± 9.3

**Yo-Yo Intermittent Recovery Test (m)** #	1	3372 ± 385	2285 ± 724	3470 ± 158	3115 ± 671
	2	3103 ± 504	1873 ± 689		2693 ± 810
	3	3408 ± 254	2168 ± 569		2788 ± 774
	4	2440 ± 198	2123 ± 689		2193 ± 617

	**Total for unions**	3285 ± 435	2176 ± 659	3470 ± 158	2910 ± 750

**Estimated VO** **_2max_** ** mL·min** **^−1^** **·kg** **^−1^** #	Level 1	68.3 ± 4.9	52.2 ± 10.2	71.6 ± 4.1	64.9 ± 9.9
	Level 2	64.8 ± 6.5	46.4 ± 9.9		58.7 ± 11.7
	Level 3	68.8 ± 3.2	50.7 ± 8.1		59.8 ± 11.2
	Level 4	56.0 ± 2.8	49.9 ± 9.5		51.3 ± 8.7

	**Total for unions**	67.2 ± 5.6	50.7 ± 9.3		71.6 ± 4.1

**Illinois Agility Test (s)** †*#	Level 1	16.0 ± 0.9	17.5 ± 1.3	16.8 ± 0.6	16.6 ± 1.1
	Level 2	16.5 ± 1.1	16.6 ± 0.6		16.5 ± 0.9
	Level 3	15.8 ± 1.2	24.3 ± 13.8		20.1 ± 10.3
	Level 4	17.6 ± 0.4	18.4 ± 2.0		18.2 ± 1.7

	**Total for unions**	16.2 ± 1.0	18.9 ± 6.2	16.8 ± 0.6	17.2 ± 4.0

**Push-up (reps)**	Level 1	38 ± 15	46 ± 16	49 ± 10	42 ± 15
	Level 2	28 ± 17	50 ± 5		35 ± 17
	Level 3	42 ± 20	31 ± 15		37 ± 1
	Level 4	22 ± 6	35 ± 15		33 ± 15

	**Total for unions**	36 ± 16	41 ± 16	49 ± 10	39 ± 16

**Plank (s)** #	Level 1	159 ± 30	123 ± 32	180 ± 0	155 ± 33
	Level 2	125 ± 43	140 ± 45		130 ± 42
	Level 3	177 ± 6	106 ± 41		141 ± 47
	Level 4	147 ± 47	110 ± 51		119 ± 50

	**Total for unions**	156 ± 34	119 ± 40.0	180 ± 0	146 ± 40

**Broad Jump (m)**	Level 1	2.2± 0.3	2.1 ± 0.4	2.0 ± 0.2	2.1 ± 0.3
	Level 2	2.0 ± 0.2	2.2 ± 0.2		2.1 ± 0.2
	Level 3	2.2 ± 0.2	2.0 ± 0.3		2.1 ± 0.2
	Level 4	1.8 ± 0.1	2.1 ± 0.3		2.0 ± 0.3

	**Total for unions**	2.1 ± 0.3	2.1 ± 0.3	2.0 ± 0.2	2.1 ± 0.3

Data are expressed as mean ± SD.

† Interaction between qualification levels and union affiliations (p<0.0001);

* Significance difference across Qualification levels (p<0.05);

# Significant difference across Unions (p<0.05)
